# Transcriptomic analysis of *Anopheles gambiae* from Benin reveals overexpression of salivary and cuticular proteins associated with cross-resistance to pyrethroids and organophosphates

**DOI:** 10.1186/s12864-024-10261-x

**Published:** 2024-04-06

**Authors:** Helga Saizonou, Lucy Mackenzie Impoinvil, Dieunel Derilus, Diana Omoke, Stephen Okeyo, Nsa Dada, Claudia Corredor, Nicola Mulder, Audrey Lenhart, Eric Ochomo, Luc S. Djogbénou

**Affiliations:** 1 Tropical Infectious Diseases Research Centre (TIDRC), University of Abomey-Calavi (UAC), Abomey-Calavi, Benin; 2 Entomology Branch, Division of Parasitic Diseases and Malaria, Centers for Disease Control and Prevention, Atlanta, GA USA; 3 Kenya Medical Research Institute (KEMRI), Centre for Global Health Research (CGHR), Kisumu, Kenya; 4 School of Life Sciences, Arizona State University, Tempe, AZ USA; 5 Human, Heredity, and Health in Africa H3ABionet network, Cape Town, South Africa; 6 Regional Institute of Public Health (IRSP), Ouidah, Benin; 7 Department of Vector Biology, Liverpool School of Tropical Medicine, Liverpool, UK

**Keywords:** Insecticide resistance, *An. gambiae*, Vector surveillance, RNA-seq, Differential gene expression

## Abstract

**Background:**

Insecticide resistance (IR) is one of the major threats to malaria vector control programs in endemic countries. However, the mechanisms underlying IR are poorly understood. Thus, investigating gene expression patterns related to IR can offer important insights into the molecular basis of IR in mosquitoes. In this study, RNA-Seq was used to characterize gene expression in *Anopheles gambiae* surviving exposure to pyrethroids (deltamethrin, alphacypermethrin) and an organophosphate (pirimiphos-methyl).

**Results:**

Larvae of *An. gambiae s.s*. collected from Bassila and Djougou in Benin were reared to adulthood and phenotyped for IR using a modified CDC intensity bottle bioassay. The results showed that mosquitoes from Djougou were more resistant to pyrethroids (5X deltamethrin: 51.7% mortality; 2X alphacypermethrin: 47.4%) than Bassila (1X deltamethrin: 70.7%; 1X alphacypermethrin: 77.7%), while the latter were more resistant to pirimiphos-methyl (1.5X: 48.3% in Bassila and 1X: 21.5% in Djougou). RNA-seq was then conducted on resistant mosquitoes, non-exposed mosquitoes from the same locations and the laboratory-susceptible *An. gambiae* s.s. Kisumu strain. The results showed overexpression of detoxification genes, including cytochrome P450s (CYP12F2, CYP12F3, CYP4H15, CYP4H17, CYP6Z3, CYP9K1, CYP4G16, and CYP4D17), carboxylesterase genes (COEJHE5E, COE22933) and glutathione S-transferases (GSTE2 and GSTMS3) in all three resistant mosquito groups analyzed. Genes encoding cuticular proteins (CPR130, CPR10, CPR15, CPR16, CPR127, CPAP3-C, CPAP3-B, and CPR76) were also overexpressed in all the resistant groups, indicating their potential role in cross resistance in *An. gambiae*. Salivary gland protein genes related to ‘salivary cysteine-rich peptide’ and ‘salivary secreted mucin 3’ were also over-expressed and shared across all resistant groups.

**Conclusion:**

Our results suggest that in addition to metabolic enzymes, cuticular and salivary gland proteins could play an important role in cross-resistance to multiple classes of insecticides in Benin. These genes warrant further investigation to validate their functional role in *An. gambiae* resistance to insecticides.

**Supplementary Information:**

The online version contains supplementary material available at 10.1186/s12864-024-10261-x.

## Background

Insecticide-based vector control approaches such as insecticide-treated nets (ITNs) and indoor residual spraying (IRS) are core methods to break human-vector contact, thus reducing transmission of malaria [1]. The insecticides traditionally used in vector control fall within 4 main classes: pyrethroids, carbamates, organochlorines, and organophosphates, with pyrroles and neonicotinoids being recently repurposed from agricultural pesticides [2]. Because of this reliance on insecticide-based control methods, the emergence and spread of insecticide resistance in mosquito vectors is becoming a serious threat.

Insecticide resistance is caused by multiple mechanisms including target site insensitivity, such as knockdown resistance (*kdr*), and metabolic resistance brought about by the increased production of enzymes capable of breaking down insecticides [3]. Knockdown resistance (*kdr*) arises from mutations in the voltage-gated sodium channel gene which is a target of pyrethroid and organochlorine insecticides, while the acetylcholinesterase (*ace-1*) gene is a target of organophosphate and carbamate insecticides. Mutations such as L1014S and L1014F in the *kdr* gene [4] and the G280S mutation in the *ace-1* gene [5] cause structural changes that decrease the ability of certain insecticides to bind with their target sites. Additionally, the overexpression of genes encoding detoxification enzymes (e.g., cytochrome P450s, glutathione S-transferases and carboxylesterases) are known to contribute to metabolic resistance in mosquitoes [6, 7] and can be markers of IR in mosquito populations [8]. Because these changes resulting in IR have a genetic basis, robust sets of molecular markers could provide sensitive and timely diagnoses of insecticide resistance in mosquito populations, thereby enabling the implementation of effective insecticide resistance management (IRM) strategies.

Next-generation sequencing approaches such as RNA-seq and whole genome sequencing (WGS) enable the understanding of the genetic variations that result in changes to mosquito biology and behavior in response to environmental factors, including insecticide pressure. Transcriptomic analyses allow for the investigation of gene expression and polymorphic variations associated with specific phenotypes [9, 10], as well as the identification of candidate genes related to resistance to a specific insecticide or multiple insecticides [9, 11, 12]. A recent study of the malaria vector *Anopheles arabiensis* found multiple highly overexpressed genes related to cuticular-associated proteins and salivary gland proteins associated with pyrethroid and organophosphate resistance, suggesting roles of these lesser-understood gene groups in cross-resistance [12].

**Fig. 1:**
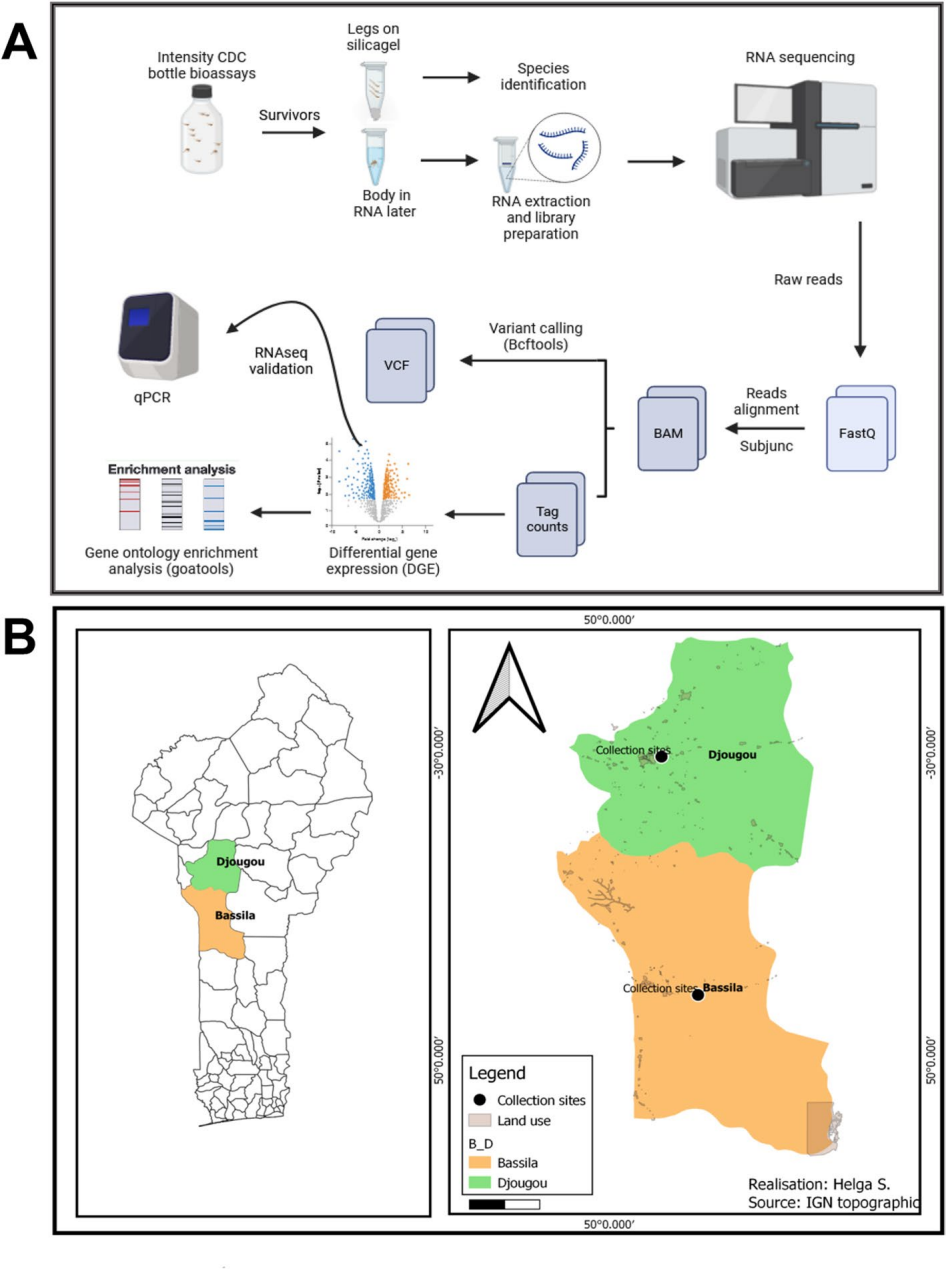
Experimental design and study sites. Panel A shows the experimental workflow. Panel B depicts the map of Benin Republic showing the two study sites, Djougou and Bassila. The black dots correspond to the collection while the land use represents where habitats are located

The objective of this study was to use transcriptomic data to identify candidate genes associated with insecticide resistance in *An. gambiae s.s* collected from Djougou and Bassila, two sites in Benin with differing levels of resistance to pyrethroid and organophosphate insecticides (Fig. 1).

## Results

### Resistance profiles of *an. Gambiae* from Bassila and Djougou

**Fig. 2:**
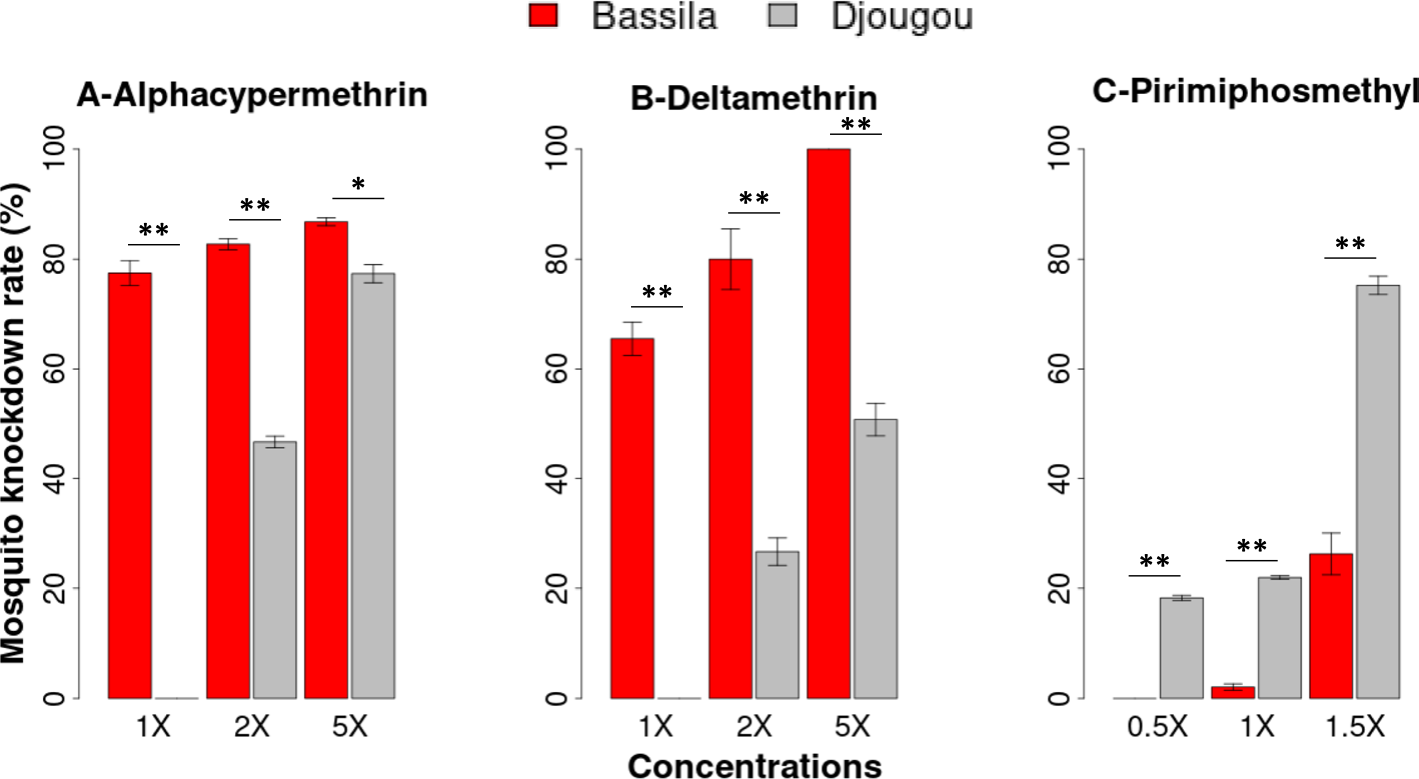
Phenotypic insecticide resistance profiles of *Anopheles gambiae* from Bassila (red) and Djougou (gray), Benin. The average mortalities of mosquitoes exposed to alphacypermethrin, deltamethrin and pirimiphos-methyl at 30 minutes are shown as percentages on the y-axis with 95% confidence intervals. * *P* < 0.05 and ** *P* < 0.005

Intensity CDC bottle bioassays were conducted on 4-to-5-day old mosquitoes from Bassila and Djougou (Fig. 1). The mortality of the mosquitoes in each insecticides bioassay is presented in Figure 2 and Additional file 1. There was a significant difference in the mortality between mosquitoes from Bassila and Djougou (*P* < 0.05) with those from Djougou being more resistant to alphacypermethrin and deltamethrin than Bassila. There was no mortality to 1X alphacypermethrin or deltamethrin in mosquitoes sampled from Djougou, improving to 46.7% (sd: 1.06) and 26.68% (sd: 2.5) for 2X doses of the two insecticides, respectively. Mortality was 77.5% (sd: 2.28) for alphacypermethrin and 65.5% (sd: 3.03) for deltamethrin in Bassila at 1X improving to 82.8% (sd: 0.1) and 80% (sd: 5.54) at 2X, respectively. At 5X exposure, the average percent mortality in Djougou was 77.4% (sd: 1.65) and 50.8% (sd: 2.98) for alphacypermethrin and deltamethrin, respectively, and 100 and 86.8% (sd: 0.72) in Bassila (Additional file 1).

In contrast to the pyrethroids, mosquitoes from Bassila were more resistant than mosquitoes from Djougou when tested against pirimiphos-methyl. The mortality following 1X exposure was 22.0% (sd: 0.31) in Djougou and 2.1% (sd: 0.58) in Bassila. At 1.5X exposure, the mortality was 75.2% (sd: 1.66) in Djougou and 26.3% (sd: 3.81) in Bassila (Additional file 1).

### RNA-Seq data quality control and mapping

The following sets of surviving mosquitoes were sequenced: from Bassila; exposed to 1X deltamethrin or 1.5X pirimiphos-methyl; from Djougou; surviving 2X alphacypermethrin, 5X deltamethrin or 1X pirimiphos-methyl along with unexposed mosquitoes from both localities and mosquitoes form the susceptible Kisumu *An. gambiae* reference strain. Raw reads generated ranged from 45 to 112 million for mosquitoes from Bassila, 51-108 million for mosquitoes from Djougou and 77-102 million for the susceptible Kisumu strain mosquitoes. After filtering, more than 98% of the reads were retained in all experiments and mapped to the reference genome of *Anopheles gambiae* PEST (VectorBase release 48). The percentage of reads mapped to the reference genome ranged between 61 and 70.6% for Bassila, 51.4 and 76% for Djougou and 69.2 and 75% for the susceptible Kisumu strain (Additional file 2), and 70 and 79% of the alignments (read pairs) were successfully assigned to the exonic features of the gene set AgamP4 (Additional file 3). The mapping rate reported here included only uniquely mapped reads, as the multi-mapped reads are not relevant for differential gene expression analysis. The relatively low uniquely mapped reads suggest that RNA-Seq of *Anopheles* may result in a large number of multi-mapped reads, likely due to the high level of repeat elements and short Illumina reads. However, similar percentages were observed in another study [12].

### Principal component analysis

To evaluate the level of similarity between mosquito strains and biological replicates, Principal component analysis (PCA) was performed on the normalized RNA-Seq data. The PCA analysis revealed that 30.44% of the total variation could be explained by PC1, while 22.1% could be explained by PC2 (Additional file 4). The RNA-seq libraries were grouped based on biological replicates and insecticide susceptibility status, validating the RNA-Seq quality and highlighting the distinct rearing histories of the mosquito populations. The analysis revealed two main clusters: 1) the insecticide-susceptible population (Kisumu), and 2) mosquitoes collected from the field, encompassing both insecticide-resistant and unexposed mosquitoes from Djougou and Bassila (Additional file 4).

### Differential gene expression analysis

**Fig. 3:**
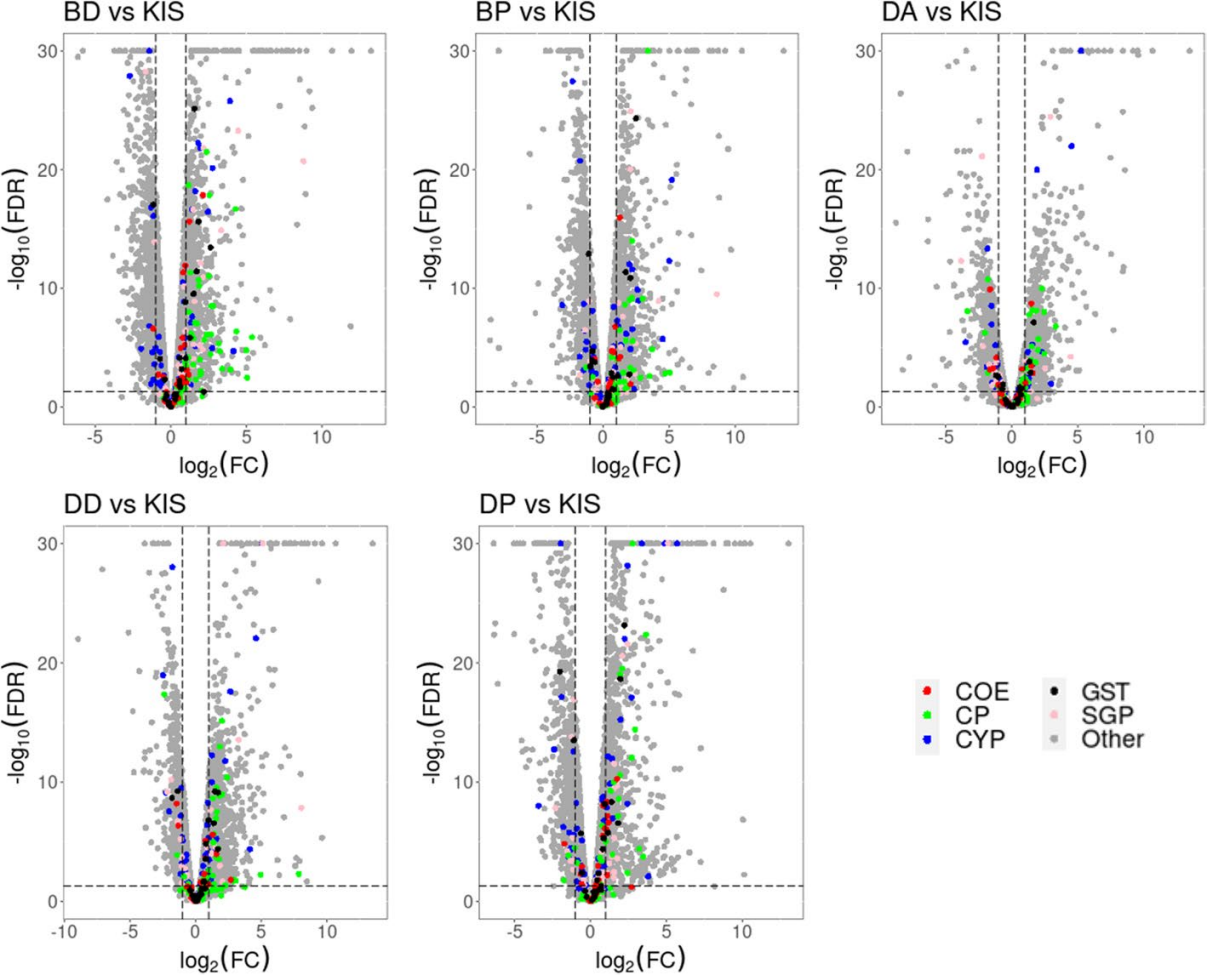
Gene expression profiles of resistant *Anopheles gambiae* from Bassila and Djougou, Benin. Volcano plots of the gene expression profiles based on mosquitoes resistant to alphacypermethrin, deltamethrin or pirimiphos-methyl when compared to the susceptible An. gambiae Kisumu strain. The gene expression level is plotted on the x-axis while the statistical significance is shown on the y-axis as log10 of the correlated *p* value. Panel (BD vs KIS) represents Bassila mosquitoes resistant to deltamethrin against Kisumu, (BP vs KIS) represents Bassila mosquitoes resistant to pirimiphos-methyl against Kisumu, (DD vs KIS) represents Djougou mosquitoes resistant to deltamethrin against Kisumu, (DP vs KIS) represents Djougou mosquitoes resistant to pirimiphos-methyl against Kisumu and (DA vs KIS) represents Djougou mosquitoes resistant to alphacypermethrin against Kisumu. Key gene families are indicated: in red (COE: carboxylesterases), blue (CYP: cytochrome P450s), pink (SGP: salivary gland proteins), green (CP: cuticular proteins) and purple (GST: glutathione-S-transferases) while other genes were represented in gray

EdgeR was used to perform differential gene expression (DGE) analysis between the resistant field mosquitoes and the laboratory susceptible mosquitoes (R-S): (DA vs KIS; DD vs KIS; DP vs KIS; BD vs KIS; BP vs KIS); the unexposed field mosquitoes and the laboratory susceptible mosquitoes (C-S): (DU vs KIS; BU vs KIS) and between the resistant field mosquitoes and the unexposed field mosquitoes (R-C): (DA vs DU; DD vs DU; DP vs DU; BD vs BU; BP vs BU). A fold-change (FC) > 2 and a false discovery rate (FDR) < 0.01 were used to identify differentially expressed genes (Fig. 3). The DGE results are summarized in Table 1 and Additional file 5.

**Table 1:** Differential gene expression summary for alphacypermethrin, deltamethrin and pirimiphos-methyl

*Insecticide*	*Site*	*Condition*	Number of genes tested	DE genes (|FC| > 2 & adjP< 0.05)		DE genes (|FC| > 2 & adjP< 0.01)	
				UP	Down	UP	Down
*Alphacypermethrin*	*Djougou*	*DA* vs *DU (R-C)*	9743	31	30	13	12
		*DA* vs *KIS (R-S)*	10,041	949	860	850	755
*Deltamethrin*	*Bassila*	*BD* vs *BU (R-C)*	9920	41	65	28	47
		*BD* vs *KIS (R-S)*	10,146	966	819	943	805
	*Djougou*	*DD* vs *DU (R-C)*	9937	68	24	45	17
		*DD* vs *KIS (R-S)*	10,154	887	573	822	555
*Pirimiphos-methyl*	*Bassila*	*BP* vs *BU (R-C)*	9740	12	50	8	39
		*BP* vs *KIS (R-S)*	10,080	968	776	926	758
	*Djougou*	*DP* vs *DU (R-C)*	9571	94	114	55	81
		*DP* vs *KIS (R-S)*	9979	953	764	932	750
*Unexposed*	*Bassila*	*BU* vs *KIS (C-S)*	9999	886	696	863	686
	*Djougou*	*DU* vs *KIS (C-S)*	10,014	757	609	698	576

• *DA *Djougou mosquitoes surviving exposure to alphacypermethrin

• *DD *Djougou mosquitoes surviving exposure to deltamethrin

• *DP *Djougou mosquitoes surviving exposure to pirimiphos-methyl

• *DU *Djougou mosquitoes unexposed

• *BD *Bassila mosquitoes surviving exposure to deltamethrin

• *BP *Bassila mosquitoes surviving exposure to pirimiphos-methyl

• *BU *Bassila mosquitoes unexposed

### Differential gene expression associated with alphacypermethrin resistance

Genes associated with alphacypermethrin resistance were derived from three different comparisons using mosquitoes from Djougou. A total of 1274, 1605 and 25 genes were significantly differentially expressed in the C-S, R-S and R-C comparisons, respectively (Table 1). Four upregulated genes were shared between the three comparison sets, with only one characterized protein belonging to the cuticular protein RR-1 family 75 (Additional file 6-A). Comparing the R-S and C-S groups, genes overexpressed in both sets are shown in Additional file 7. Interestingly, one carboxylesterase (COE22933), six cuticular proteins (CPR76, CPR75, CPR16, TWDL1, CPR81, and CPAP3-A1b), two members of the cytochrome P450 family (CYP4D17 and CYP12F2) and two salivary gland proteins (salivary secreted peptide (AGAP013060) and kDa salivary (AGAP004316)) showed higher fold changes in the R-S comparison compared to the C-S comparison. Additional genes such as DE-cadherin-like isoform X1 (AGAP029696), COX2, TEP1, and ESP also had a higher fold change in the R-S group (Additional file 7).

Importantly, a comparison between the R-S and R-C groups (Additional file 8) showed that a cuticular protein (CPR75) and a carboxylesterase (COEunkn) were differentially expressed and shared among both comparisons.

The R-S comparisons showed an overexpression of three cuticular proteins (CPAP3-A1c, CPAP3-A1a, and CPAP3-D), two salivary gland proteins (salivary secreted mucin 3 (AGAP009473), secreted salivary gland (AGAP001989)), three carboxylesterases (COE12O, COEunkn, and COEBE3C), three cytochrome P450s (CYP12F3, CYP306A1, and CYP4G16), and one odorant-binding protein (AGAP012867) that were not significantly expressed in the C-S set of genes (Additional file 7). Additionally, the top 10 genes (FC ~ 11.2-380.5) overexpressed in the R-S set of genes were genes unrelated to detoxification enzymes such as autophagy 12-like (AGAP012847), DE-cadherin-like isoform X1 (AGAP029698), or NADH dehydrogenase subunit 6 (AGAP028386) (Additional file 7).

### Differential gene expression associated with deltamethrin resistance

In Bassila, 1549, 1748 and 75 genes were significantly differentially expressed in C-S, R-S and R-C comparisons, respectively (Table 1). No DEGs were shared between the three groups (Additional file 6-B). In Djougou, the number of DEGs significantly differentially expressed in the C-S, R-S and R-C comparisons were 1274, 1377 and 62 genes, respectively (Table 1). Unlike in Bassila, five DEGs were upregulated and shared between the three groups (Additional file 6-C). Among them, two had retrievable annotations and were related to SERAC1 isoform X1 (AGAP011044) and serine protease (CLIPB5).

A total of 1240 and 887 DEGs were shared between the R-S and C-S groups from Bassila and Djougou, respectively. DEGs with notably higher expression in Bassila within the R-S group compared to the C-S group included 35 detoxification genes. The difference in expression between the R-S and the C-S groups showed that cuticular and salivary gland proteins accounted for some of the most over expressed (CPCFC1, CPR125, CPR140, D7r2, SG7, SG3) genes. In Djougou, DEGs included 14 detoxification genes, 7 cuticular proteins (CPAP3-A1b, CPR76, TWDL1, CPAP3-B, CPR81, CPR75, and CPAP3-E) and one salivary gland protein (SG2), suggesting the importance of these gene families to the insecticide resistant phenotype**.** Additional DEGs with higher expression in the R-S group compared to the C-S group included TEP9, GNBPB4, and indirect flight muscle (AGAP011514) in Bassila, and CLIPB5, TEP4, ESP, CLIPC7, and SERAC1 isoform X1 (AGAP011044) in Djougou (Additional file 7).

Comparing the R-S and R-C groups, both sets shared CYP6Z3 in Bassila, while CPR9, CPR144 and CYP4H24 were shared in Djougou. Additionally, only the angiopoietin-like salivary protein (AGAP007041) in Bassila and CYP314A1 in Djougou were overexpressed solely in the R-C group. Other genes such as fibrinogen A (AGAP011228), CLIPB12, PPO6, and PGRPLB were overexpressed in the Djougou R-C group. (Additional file 8).

**Fig. 4:**
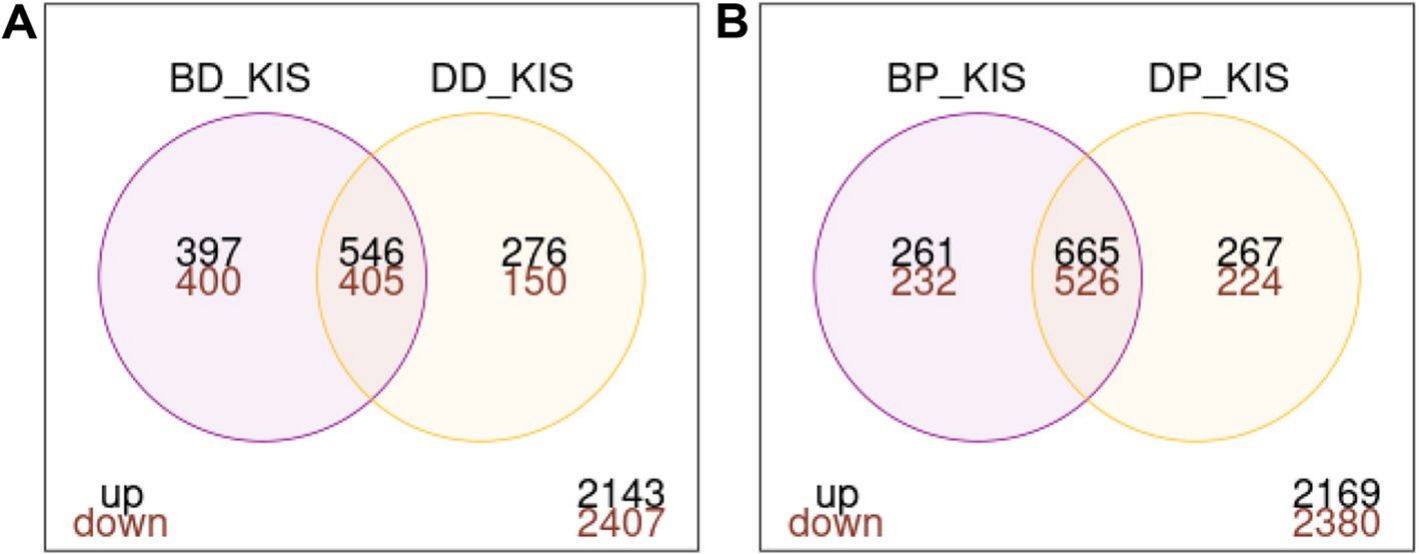
Venn diagrams showing differentially expressed genes. Panel A represents genes differentially expressed in Bassila and Djougou mosquitoes resistant to deltamethrin; B represents genes differentially expressed in mosquitoes from both sites resistant to pirimiphos-methyl. Each Venn diagram section shows the number of differentially expressed genes meeting each set of conditions and the P -values were adjusted for multiple testing based on FDR < 0.01 and FC > 2

**Fig. 5:**
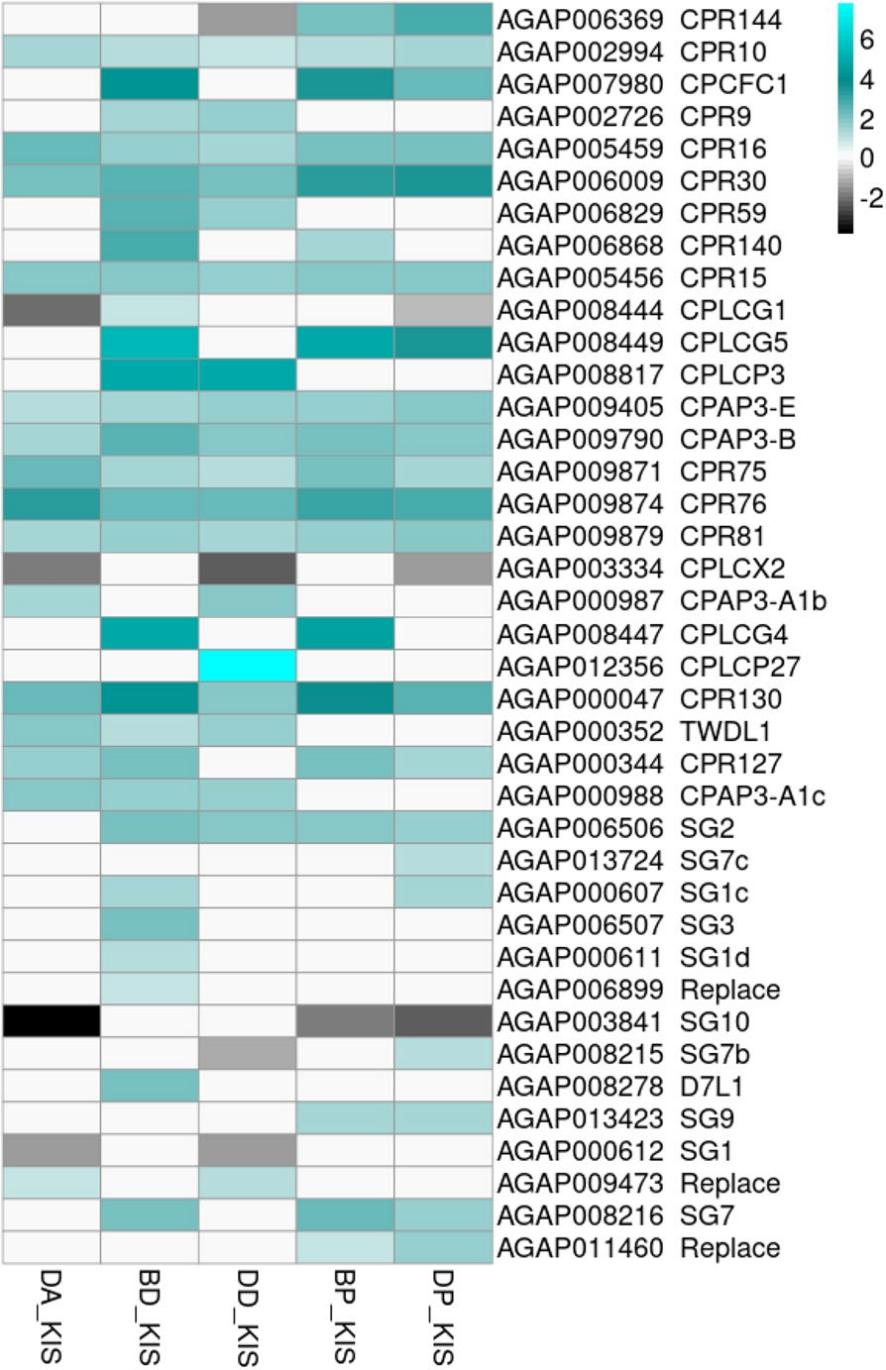
Heatmap showing cuticular and salivary gland proteins genes expressed in each R-S comparison. The heatmap shows log_2_fold-change values relative to the susceptible strain Kisumu (KIS) on a black-cyan scale. The cyan color indicates overexpression. Genes represented in this plot are CPs and SGPs shared among all R-S comparisons as well as those specifically expressed in either pyrethroids R-S or organophosphates R-S comparisons. DP_KIS = Djougou mosquitoes resistant to pirimiphos-methyl vs. the susceptible strain Kisumu, DD_KIS = Djougou mosquitoes resistant to deltamethrin vs. the susceptible strain Kisumu, DA_KIS = Djougou mosquitoes resistant to alphacypermethrin vs. the susceptible strain Kisumu, BP_KIS = Bassila mosquitoes resistant to pirimiphos-methyl vs. the susceptible strain Kisumu and BD_KIS = Bassila mosquitoes resistant to deltamethrin vs. the susceptible strain Kisumu

**Fig. 6:**
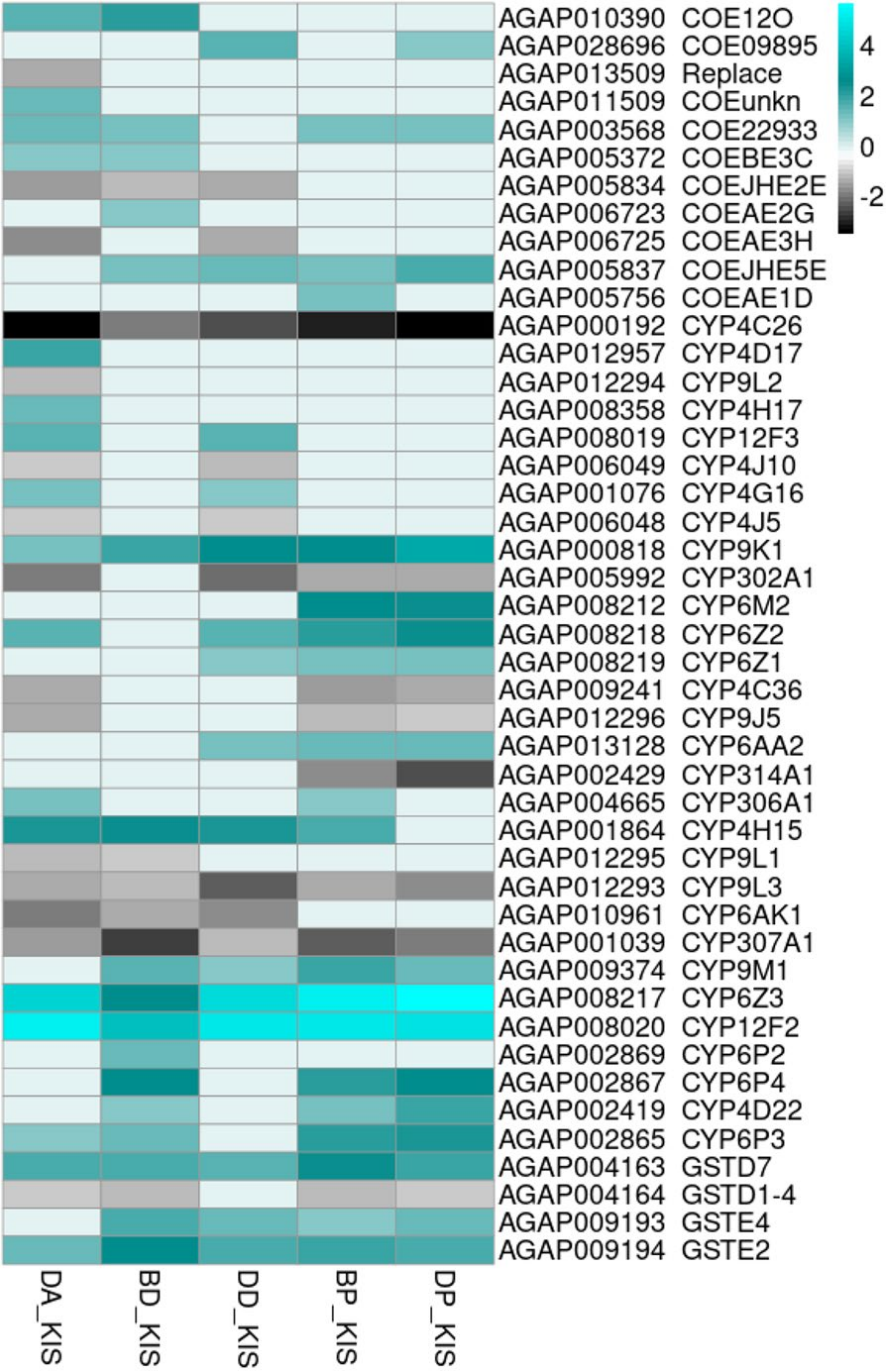
Heatmap showing cytochrome P450s, COEs and GSTs genes expressed in each R-S comparison. The heatmap shows log_2_fold-change values relative to the susceptible strain Kisumu (KIS) on a black-cyan scale. The cyan color indicates overexpression. Genes represented in this plot are COEs, CYPs and GSTs shared among all R-S comparisons as well as those specifically expressed in either pyrethroids R-S or organophosphates R-S comparisons. DP_KIS = Djougou mosquitoes resistant to pirimiphos-methyl vs. the susceptible strain Kisumu, DD_KIS = Djougou mosquitoes resistant to deltamethrin vs. the susceptible strain Kisumu, DA_KIS = Djougou mosquitoes resistant to alphacypermethrin vs. the susceptible strain Kisumu, BP_KIS = Bassila mosquitoes resistant to pirimiphos-methyl vs. the susceptible strain Kisumu and BD_KIS = Bassila mosquitoes resistant to deltamethrin vs. the susceptible strain Kisumu

Focusing on DEGs within the R-S group, a total of 951 genes were commonly differentially expressed in deltamethrin survivors from both the Bassila and Djougou populations relative to the susceptible strain (Fig. 4-A). The upregulated genes included cuticular proteins (CPLCP3, CPR130, CPR30, CPR59, CPAP3-B, CPR76, and TWDL1), a salivary gland protein (SG2) (Fig. 5), some metabolic genes (COEJHE5E, CYP4H15, CYP12F2, CYP6Z3, CYP9K1, CYP9M1, GSTE2, GSTD7, and GSTE4) (Fig. 6), and an odorant binding protein (OBP47). Interestingly, the detoxification genes CYP12F2 and CYP6Z3 exhibited higher transcription activity in deltamethrin survivors from Djougou (FC = 33.6 & 24.3, respectively) than in those from Bassila (FC = 15.1 & 6.7, respectively), suggesting their potential association with the differing intensities of resistance between the two field populations (Additional file 9).

### Differential gene expression associated with pirimiphos-methyl resistance

Differentially expressed genes in Bassila included 1549, 1684 and 47 genes in C-S, R-S and R-C respectively **(**Table 1). In Djougou, 1274, 1682 and 136 were differentially expressed in C-S, R-S and R-C, respectively (Table 1). In Bassila, a total of 1220 DEGs were shared between the R-S and C-S groups (Additional file 6-D). Some of them exhibited higher fold change of expression in the R-S group than in the C-S group, including 20 detoxification genes. SG7 and some members of the cytochrome P450 family (CYP9M1, CYP6Z2, CYP6P3, CYP6Z3) were more overexpressed in the R-S group than C-S group (Additional file 7). In Djougou, 982 DEGs were shared between the R-S and C-S comparisons (Additional file 6-E). From these DEGs, those that exhibited higher fold changes included cuticular proteins (CPR30, CPR76, CPR16, CPLCG1, CPR81, CPLCX2, and CPAP3-E), cytochrome P450s (CYP6M2, CYP6P3, CYP6Z3, CYP4D22, and CYP9K1), and a carboxylesterase (COE22933). Other DEGs with retrievable annotations were two trypsin-related proteases (TRYP7, and AGAP012842), a glycine-rich cell wall structural-like (AGAP008892) and others (Additional file 7).

Comparing the R-S and R-C groups, no metabolic genes were shared among the two comparisons in Bassila, while CYP6Z3 and GSTD11 were shared in Djougou. Additionally, two carboxylesterases PPO6, PPO9, PGRPLB, and fibrinogen A (AGAP011228) were overexpressed in only the R-C group from Djougou (Additional file 8).

Focusing on the DEGs in the R-S group, a total of 1191 were differentially expressed in the pirimiphos-methyl survivors from both Bassila and Djougou when compared to the susceptible strain **(**Fig. 4-B). These shared DEGs included cuticular proteins (CPLCG5, CPR130, CPR30, CPCFC1, CPR76, CPR75, CPAP3-B, CPR127, CPR15, CPR16, and CPR144), salivary gland proteins (SG7, SG2, SG9) (Fig. 5), some metabolic genes (COE22933, COEJHE5E, CYP6Z3, CYP12F2, CYP6M2, CYP9K1, CYP6P4, CYP6P3, CYP6Z2, GSTD7, GSTE2, and GSTE4) (Fig. 6), and two odorant binding proteins (OBP47, OBP26) (Additional file 9).

### Genes associated with resistance to multiple insecticides

**Fig. 7:**
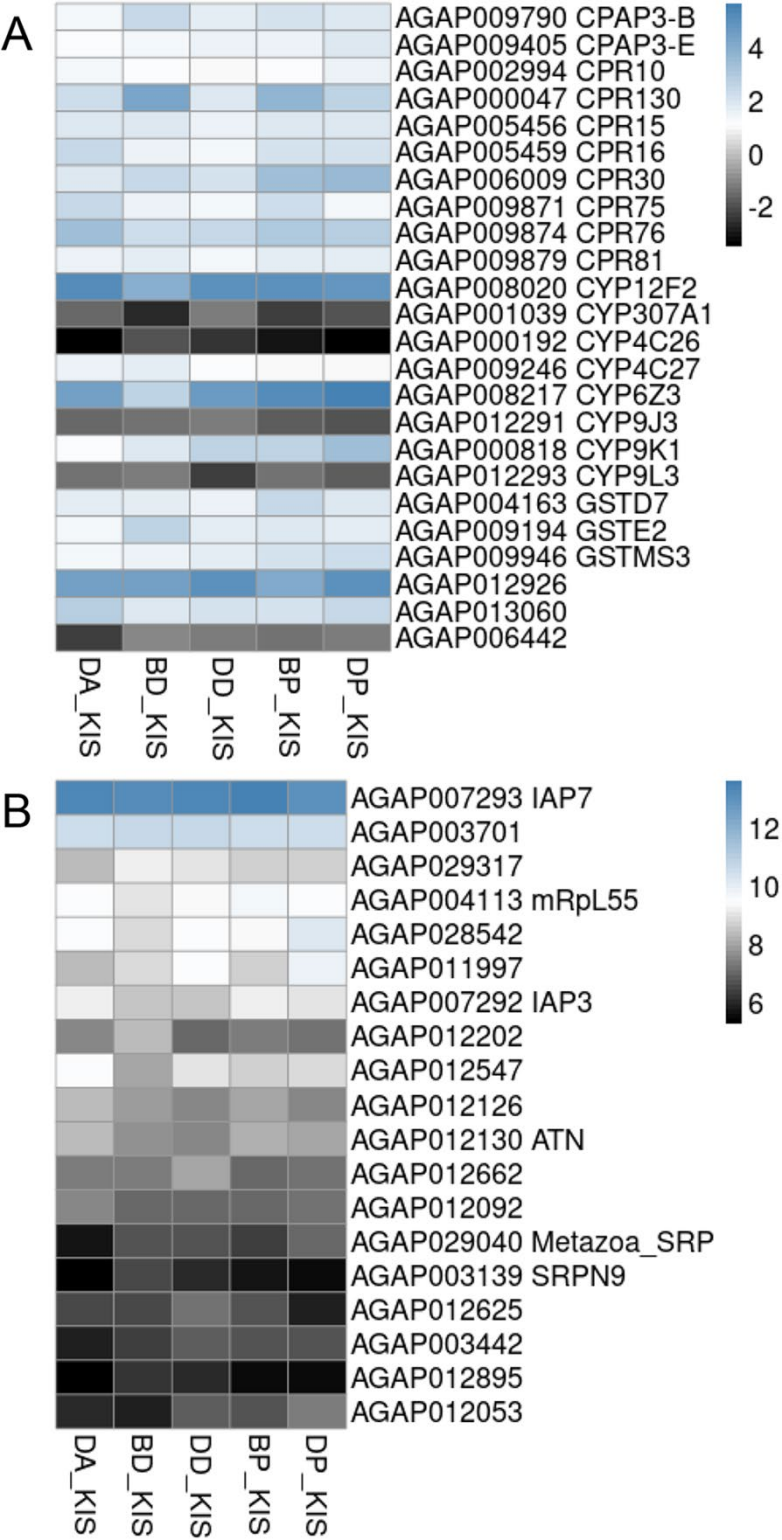
Differentially expressed genes associated with resistance to multiple insecticides. Panel A shows a heatmap underlining the log2-fold change (log2FC) expression of the 24 detoxification genes differentially expressed in all the R-S comparisons. Panel B shows the log2-fold change (log2FC) expression of the top 20 genes differentially expressed in all the R-S comparisons. A black-blue scale was used with the color blue indicating over-expression

A total of 500 DEGs were shared by mosquitoes that survived exposure to either alphacypermethrin, deltamethrin or pirimiphos-methyl (Additional file 10). Among those genes, were cuticular proteins (CPAP3-B, CPAP3-E, CPR10, CPR130, CPR15, CPR16, CPR30, CPR75, CPR76, and CPR81), cytochrome P450s (CYP12F2, CYP307A1, CYP4C26, CYP4C27, CYP6Z3, CYP9J3, CYP9K1, and CYP9L3), glutathione S- transferases (GSTD7, GSTE2, and GSTMS3) and some uncharacterized salivary gland proteins (Fig. 7-A, Additional file 11). Other genes (FC ~ 5.93-13.25) related to inhibitor of apoptosis, zinc finger 593, ribosomal mitochondrial, flotillin − 2, acyl-thioester, allatropins, serine protease inhibitor (SRPN9) were among the top 20 genes over-expressed in all R-S comparisons (Fig. 7-B).

### Non-synonymous target site mutations

**Fig. 8:**
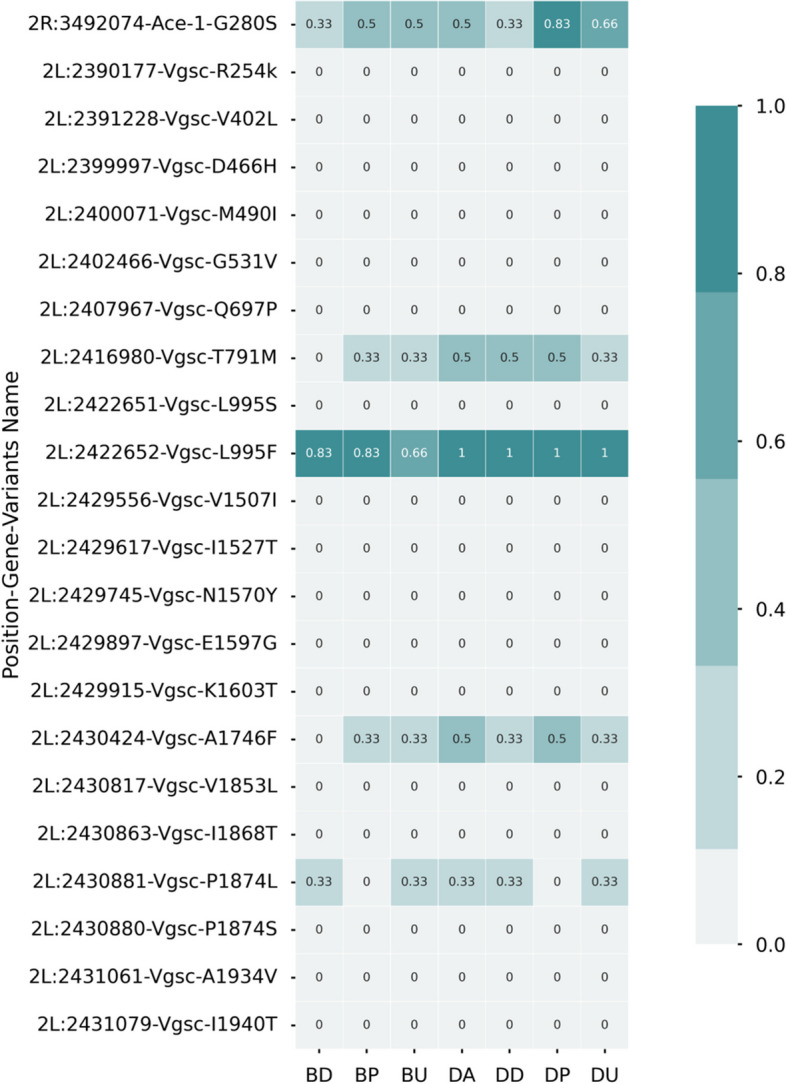
Non-synonymous target site mutations. The heatmap shows on a gray-teal scale (teal = 1) the overall average allele frequency observed in each group. Only mutations in the Ace-1 gene (G280S) and mutations L995F, T791M, A1746F and P1874L in the Vgsc gene were observed within the data sets. All other mutations were not detected even with reads spanning those genomic locations

Target site mutations on the *Ace-1* and in the *kdr* genes were identified through the analysis of single nucleotide polymorphisms (SNPs). The analyzed samples were composed of pools of seven mosquitoes each, and a single population included three replicates (pools). The pools also contained all non-phenotyped mosquitoes (unexposed). The frequency at which polymorphisms appeared in a population was derived from the depth coverage at each position with the contribution of each mosquito in the pools. The G280S mutant allele frequency in the *Ace-1* gene was 50% for Bassila compared to 66% for Djougou (Fig. 8). For the *kdr* gene, among variants of interest highlighted in a previous study [13], only mutations at positions L995F, T791M, A1746F, and P1874L were detected. The L995F mutation frequency was 33% in Bassila and 100% in Djougou, while the mutant allele frequency for the variants T791M, A1746H and P1874L were of 33% in both populations Fig. 8*.*

### Gene ontology annotation and enrichment analysis

Gene ontology enrichment (GOE) analysis was conducted on differentially expressed genes (up and downregulated) for all R-S comparisons. Gene ontologies are classified into three classes: biological process (BP), molecular function (MF) and cellular component (CC) (Additional file 12).

Among BP GO terms, terms related to proton transport (GO:0015986), aerobic respiration (GO:0009060) and mitochondrial electron transport (GO:0006123, GO:0006120) were shared in the BD vs KIS, BP vs KIS, DD vs KIS and DP vs KIS comparisons. Furthermore, GO terms associated with glycolytic processes (GO:0006096) were enriched in BP vs KIS and DP vs KIS, while the carbohydrate metabolic processes (GO:0005975) were enriched in BD vs KIS and DD vs KIS (Additional file 13). Considering the CC GO terms, terms associated with mitochondrial respiratory chain complex I (GO:0005747) III (GO:0005750) and IV (GO:0005751) were enriched in BD vs KIS, BP vs KIS, DD vs KIS and DP vs KIS with mitochondrial proton-transporting ATP synthase complex and coupling factor F(o) (GO:0000276) enriched in only the BP vs KIS and DP vs KIS (Additional file 13). Concerning terms related to MF, macromolecular complex binding related terms (GO:0044877) and sulfur cluster binding (GO:0051539) were enriched in all R-S comparisons except DA vs KIS. Hydrogen ion transmembrane transporter activity (GO:0015078) was enriched in both BP and DP vs KIS, highlighting its strong correlation with resistance to pirimiphos-methyl (Additional file 13)*.*

### RNA-Seq data validation using quantitative PCR

**Fig. 9:**
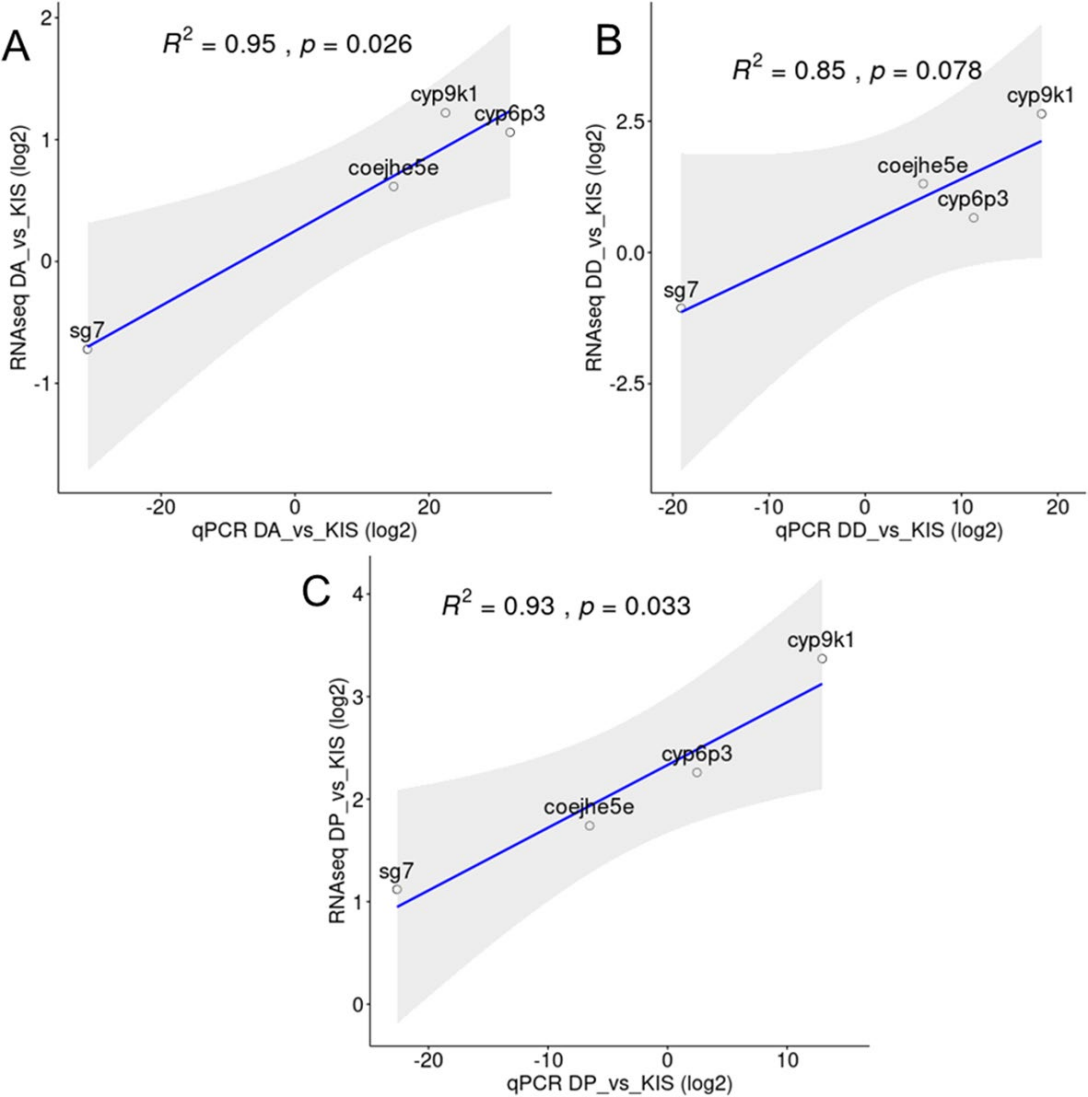
Correlation of expression levels between qRT-PCR and RNA-Seq data. The different panels show the correlation between qRT-PCR results and RNA-Seq data for the four selected genes in each sample. **A**) Djougou mosquitoes surviving exposure to alphacypermethrin compared to Kisumu, **B**) Djougou mosquitoes surviving exposure to deltamethrin compared to Kisumu, **C**) Djougou mosquitoes surviving exposure to pirimiphos-methyl compared to Kisumu

The expression patterns of four genes (SG7, CYP9K1, CYP6P3 and COEJHE5E) were validated in relation to two housekeeping genes (40S ribosomal protein S7; RPS7 and Actin5c) (Additional file 14, Fig. 9). Most of the qPCR results supported the directionality of the expression level changes observed after RNA sequencing (*P* < 0.05 and R^2^ > 90) (Fig. 9). Nevertheless, for Djougou mosquitoes exposed to deltamethrin, R^2^ was equal to 85 with *P* = 0.078 (Fig. 9), which could be due to an overestimation in the RNA-Seq data.

## Discussion

In this study, we analyzed the gene expression profiles of two *An. gambiae* populations following exposure to key insecticides used for malaria vector control. In addition to detecting differential expression of genes encoding detoxification enzymes, genes coding for salivary gland and cuticular proteins were also overexpressed in mosquitoes from both study sites.

The two mosquito populations displayed variable levels of resistance to alphacypermethrin, deltamethrin and pirimiphos-methyl. A higher intensity of resistance to both alphacypermethrin and deltamethrin was observed in Djougou than in Bassila despite both being located in Donga district in northern Benin. Such heterogeneity in the levels of insecticide resistance despite relative geographic proximity has also been described elsewhere [14, 15]. The high pyrethroid resistance detected in the Djougou population could be related to the intensive use of pyrethroids in vector control by the NMCP which has intensified ITN distribution every 3 years since 2008 in Djougou [16]. In addition, there is intensive use of carbamates, organophosphates and pyrethroid pesticides for agricultural activities in Donga district [16, 17]⁠. In contrast, in Bassila, although also an agricultural site, no NMCP vector control interventions have been distributed to date. The lower alphacypermethrin and deltamethrin resistance in mosquitoes from this locality might thus be related to reduced selective pressure. For this reason, the increased resistance to pirimiphos-methyl is surprising as it was higher than that of Djougou.

Cytochrome P450s are naturally abundant in insects [18] and their primary function is to metabolize pheromones and xenobiotics [18–21]. In the Djougou mosquitoes that survived alphacypermethrin exposure, two members of this family, CYP4H17 and CYP4D17, were overexpressed. Both genes have been found to be overexpressed in mosquitoes resistant to permethrin [22, 23] with an overexpression of CYP4D17 in mosquitoes exposed to 0.05% deltamethrin [23]. Their over-expression only in mosquitoes resistant to alphacypermethrin here is quite interesting given that they were not overexpressed in deltamethrin resistant mosquitoes from the same population. Further, CYP4G16 and CYP12F3 were overexpressed in Djougou mosquitoes that survived both alphacypermethrin and deltamethrin exposure. Overexpression of CYP4G16 has been demonstrated to be important in cuticular hydrocarbon content enrichment and thus reducing the uptake of pyrethroid insecticides [23, 24]. On the other hand, over-expression of CYP12F3 has not yet been associated with insecticide resistance. Functional validation of these four genes associated with the high intensity of resistance recorded to these class two pyrethroids would confirm the role played in conferring resistance.

Several CYP450s were found to be commonly shared among all R-S comparisons including CYP9K1, CYP6Z3 and CYP12F2. All three were shown to be consistently overexpressed in both pyrethroid and pirimiphos-methyl resistant mosquitoes. CYP9K1 has previously been shown to be involved in the metabolism of deltamethrin and pyriproxyfen in *An. gambiae* [23, 25] and therefore its overexpression in the pyrethroid-resistant groups is not surprising⁠. Additionally, a similar study in *An. arabiensis* showed its association with resistance to both organophosphates and pyrethroids [12],⁠ consistent with our findings here of its association with resistance to both chemical classes. CYP6Z3 has previously been associated with pyrethroid metabolism [24] but here we observed its overexpression in survivors exposed to both pyrethroids and pirimiphos-methyl indicating a potential role in cross-resistance as has been reported before [26–29]. CYP12F2 has been previously associated with permethrin resistance in *An. arabiensis* [30, 31]⁠ and was associated with both pyrethroid and organophosphate resistance in our study, suggesting a role in the detoxification of both insecticide classes. Moreover, CYP6Z2, CYP9M1 and CYP4H15 were found to be overexpressed in 4 out of 5 R-S comparison groups, with the exception of BD vs KIS. The Bassila population was not as intensely resistant to pyrethroids as Djougou, which suggests that these three genes could potentially contribute to intensified pyrethroid resistance as well as pirimiphos-methyl resistance.

Other metabolic genes including three glutathione-S-transferase genes (GSTD7, GSTE2, GSTE4) and two carboxylesterase genes (COEJHE5E, COE22933) were overexpressed in all R-S comparisons. This is consistent with the understanding that GSTs contribute to resistance against multiple insecticide classes [32]⁠. They participate in the detoxification of xenobiotics and metabolize secondary products from other metabolic activities (CYP450s, COEs) [32, 33]⁠. COEJHE5E has been recently found to be overexpressed in deltamethrin and permethrin resistant *An. coluzzii* population [23], and genomic signals of resistance to deltamethrin were found around the COE22933 locus in *An. gambiae* and *An. coluzzii* [34]. A functional validation of these two genes might enhance understanding of their role in pyrethroid resistance.

In addition to metabolic genes, a wide variety of genes encoding cuticular proteins (CPs) were found to be overexpressed in R-S comparisons. Among them, CPR10, CPAP3-E, CPAP3-B, CPRd30, CPR130, CPR15, CPR16, CPR76, CPR75, and CPR81 were overexpressed in all R-S comparisons. Their mechanism of function is not well understood but alterations to the mosquito cuticle by thickening or hardening could inhibit the penetration of insecticides and other toxicants [35]. Additionally, recent studies highlighted the massive production of chitin to enhance metabolic gene functions, such as CYPs, which could lead to cuticle hardening in *Aedes aegypti* [36]. If this is the case, this mechanism would lead to cross-resistance of insecticides regardless of class. CPAP3s belong to a group of proteins that maintain the structural integrity of the cuticle [37]⁠. CPAP3s with an obstructor-E function have been found to play a role in multiple insecticide resistance in *An. gambiae* [38]*.* In this study, CPAP3-B was overexpressed in all R-S comparisons making this gene a potential candidate marker for cross-resistance. Additional cuticular genes were observed to be more specifically overexpressed in response to exposure to certain classes of insecticides: CPR9, CPR59, CPLCP3, TWDL1, CPAP3-A1b and CPAP3-A1c in response to pyrethroids and CPR144 in response to pirimiphos-methyl. The involvement of CPLCP3 in cuticle barrier formation has been previously described and its overexpression has been associated with mosquito survival following deltamethrin exposure [39]. The overexpression of CPAP3-A1b and CPAP3-A1c has also been associated with resistance to permethrin, deltamethrin or lambdacyhalothrin [40–42].

Recently, salivary gland proteins have been reported to play a potential role in insecticide resistance [12]. In this study, three salivary gland genes stood out among the overexpressed genes in resistant mosquitoes. A threonine serine-rich mucin (SG9: AGAP013423, FC ranging from 2.51 to 2.62) and a salivary cysteine-rich peptide (AGAP011460, FC ranging from 2.03 to 3.38) were overexpressed in mosquitoes resistant to pirimiphos-methyl, while salivary secreted mucin 3 (AGAP009473, FC ranging from 2.04 to 2.21) was overexpressed in Djougou mosquitoes resistant to alphacypermethrin and deltamethrin. The salivary protein SG9 is a mucin protein. The role of mucin proteins in mosquitoes remains uncharacterized to date, unlike in vertebrates (including humans). They are found in the peritrophic membrane and have been reported to only be induced in female mosquitoes after blood ingestion [43]⁠. Here, we found a slight overexpression of this gene in mosquitoes resistant to pirimiphos-methyl. It has been demonstrated that salivary gland mucin-like protein in *Drosophila* could perform an immune defense reaction [44],⁠ and for this reason, we hypothesize that SG9 could perform a similar function in protecting and defending the respiratory wall against the penetration of organophosphate molecules. In humans, for example, exposure to low-level OPs triggers increased mucin secretion in asthmatic patients [45]⁠. This might also be the case in mosquitoes, highlighting the need to further investigate mucin-like protein functions.

On the other hand, AGAP011460 and AGAP009473 are two uncharacterized genes that were both highly overexpressed in mosquitoes resistant to pirimiphos-methyl and both pyrethroids. Further characterization and functional annotation are needed to understand their involvement in insecticide resistance.

Furthermore, in mosquitoes from Djougou that were resistant to both pyrethroids, there was an overexpression of the thioester-containing protein 1 (TEP1). Genes of the TEPs family are key components of the innate immune systems of mosquitoes [34]. Specifically, TEP1 is known to inhibit the development of malaria parasite in the midgut of mosquitoes through ookinete lysis and melanization [46, 47]. The overexpression of this gene in mosquitoes that are highly resistant to pyrethroids could imply that the immune system of mosquitoes might not only suppress parasite or microbe growth in the mosquitoes but also could play a role in insecticide detoxification. Indeed, recent studies have shown the overexpression of TEP1 in mosquitoes resistant to permethrin and deltamethrin [23, 34], highlighting how the immune system could trigger insecticide resistance. Moreover, genes from the serine protease family (CLIPAs, CLIPBs, CLIPCs and CLIPEs) were over-expressed with a slight down-regulation of prophenoloxidases (PPO; PPO6, PPO9) in mosquitoes resistant to alphacypermethrin from Djougou. Through cascade reactions, CLIPC genes activate CLIPB genes which further cleave PPOs into their active form, phenoloxidase (PO) [48, 49]. The latter plays a crucial role in the mosquito immune system and is said to have, apart from melanization function, the ability to harden the insect epidermis as a response to abiotic stress [50]. As such, the activation of PO might reduce insecticide penetration, resulting in resistance in mosquitoes. Further research is needed to understand the role of genes linked to the mosquito immune system in insecticide resistance.

In addition to differential gene expression, target site mutations are important genetic markers of phenotypic insecticide resistance. Mutations such as L995F or L995S in the *kdr* gene [4] and G280S mutations in the *Ace-1* gene [5] have been reported to confer resistance to insecticides. The data on target site mutations presented here are relatively low resolution, as they were extrapolated from RNA-Seq data on pooled mosquitoes. Nevertheless, these data provide some insight into the presence of these mutations in the two populations. The mutation L995F was found in Djougou with an allele frequency of one (100%) and in Bassila with an allele frequency of 0.33 (33%). This is likely the result of evolution due to the selective pressure caused by the intensive use of pyrethroid insecticides in Djougou [16, 17]⁠. A frequency of 0.66 (66%) of the G280S mutation which can confer resistance to organophosphates was detected in Djougou. although this population was less resistant to pirimiphos-methyl than Bassila where the mutation frequency was 0.5 (50%). Previous research has shown that the G280S mutation may not be a strong predictor of resistance to all organophosphates [51]. The overexpression of the genes described above could be important contributors to the resistant phenotypes observed.

## Conclusions

The analysis of RNA-Seq data allowed us to describe differentially expressed genes and target site mutations that were associated with pyrethroid and organophosphate resistance in *An. gambiae* from two locations in Benin: Bassila and Djougou. Multiple genes, including members of the cytochrome P450 family (CYP4H17, CYP4D17 and CYP12F2), salivary gland proteins (SG9, AGAP011460 and AGAP009473) and cuticular proteins (CPR30, CPR130, CPR15, CPR16, CPR76, CPAP3-A1b and CPAP3-A1c) were found overexpressed in resistant mosquitoes after exposure to alphacypermethrin, deltamethrin, or pirimiphos-methyl. The DEGs described here are potential molecular markers of insecticide resistance that could be incorporated into Benin’s NMCP insecticide resistance surveillance and management strategy once validated.

## Methods

### Study sites and samples

*Anopheles gambiae* larvae were collected in August 2019 from Djougou (9° 42′ 29.1312″ N and 1° 39′ 58.8672″ E) and in October 2019 from Bassila (9° 0′ 23.0148″ N and 1° 39′ 50.1264″ E) (Fig. 1) from puddles, swamp areas and drains near irrigated croplands using the dipping method. Mosquito larvae were brought back to the insectary of the ‘Environnement, Gestion des Données et Formation Universitaire (EGDFU)’ unit of the Tropical Infectious Diseases Research Center (TIDRC) of Benin Republic and reared to the adult stage under insectary conditions (insecticide-free environment, 27 ± 2 °C ambient temperature, 70 ± 8% relative humidity and 12 h:12 h light:dark photoperiod). Larvae were fed ad libitum with TetraMin Baby® fish food and emerged adults were fed on 10% honey solution.

*Anopheles gambiae* from the insecticide susceptible reference Kisumu laboratory strain were reared in the insectary at the Centers for Disease Control and Prevention (CDC), Atlanta, Georgia, USA. Mosquitoes were maintained at a constant 27 ± 2 °C and 70 ± 10% humidity on a 14 h:10 h hour light:dark cycle and adults were provided 10% sucrose ad libitum.

### Insecticide resistance intensity bioassays

Insecticide resistance intensity assays using modified CDC bottle bioassays were conducted on the reared adult mosquitoes using 1X, 2X, and 5X the diagnostic doses of deltamethrin and alpha-cypermethrin and 0.5X, 1X and 1.5X the diagnostic doses of pirimiphos-methyl. Stock solutions (10X) of the diagnostic doses (1X for deltamethrin: 12.5 μg/bottle, alphacypermethrin: 12.5 μg/bottle and pirimiphos-methyl: 20 μg/bottle) were prepared by diluting technical grade insecticide in 50 mL of absolute ethanol. Lower doses were obtained by serial dilution.

For each concentration, four bottles were coated with 1 ml of insecticide solution and one control bottle was coated with absolute ethanol. Alphacypermethrin and deltamethrin coated bottles were covered to keep them protected from light and to allow evaporation of the solvent overnight while the bottles coated with pirimiphos-methyl were left covered and protected from light for 8 hours.

Approximately 10 to 25 mosquitoes aged 4–5 days and fed with 10% honey solution were released in each bottle using an aspirator. The exposure time was set to 30 min, after which the mosquitoes were removed from the bottles and sorted into “alive” and “knocked-down” groups. Mosquitoes alive after 30 minutes of exposure to insecticide were considered resistant [52, 53]. Mosquitoes from the control bottle were labeled as unexposed.

### Mosquito species identification

The legs of the resistant and unexposed mosquitoes were removed, and the rest of the body was immediately stored in RNA later. DNA extraction from the legs was performed following the protocol of Myriam and Cecile (2003) using the cetyltrimethyl ammonium bromide (CTAB) technique. Mosquito species identification was carried out to distinguish members of the *An. gambiae s.l* species complex using species-specific PCR primers for *An. gambiae s.s,* and *An. arabiensis*. The PCR mix had a total volume of 20 μl composed of 5 μl of DNA, 8*.*4 μl of H_*2*_O, 2.5 μl of reaction buffer 1x, 0.1 μl of Taq DNA polymerase*,* 1 μl of MgCl_2_, and 1 μl of each primer (Universal forward primer: *5′-GTGTGCCCCTTCCTCGATGT-3′*; *An. gambiae s.s* reverse primer:*5′-CTGGTTTGGTCGGCACGTTT-3′* and *An. arabiensis* reverse primer: *5′-AAGTGTCCTTCTCCATCCTA*). The PCR cycling conditions were: 94 °C for 5 min, followed by 25 amplification cycles (94 °C for 30 s, 72 °C for 30 s) and a final elongation step at 72 °C for 5 min. The PCR products were visualized using 1.5% agarose gels stained with 5 μl of BET. The DNA bands on the gel (390 bp for *An. gambiae s.s.* and 315 bp for *An. arabiensis*) were compared to a 1 kb reference ladder.

### RNA extraction, RNA-Seq library preparation and sequencing

Four-to-five-day-old adult nonblood-fed female mosquitoes from the susceptible Kisumu strain were killed by freezing and stored at − 80 °C until RNA extraction. Mosquitoes from the field populations unexposed to insecticides and those that survived to the highest dose of each insecticide were stored in RNAlater and shipped to the Entomology Branch laboratory at the CDC, Atlanta, USA, for RNA extraction, library preparation and sequencing.

RNA extraction was conducted using the Arcturus PicoPure RNA isolation kit (Life Technologies, USA) according to the manufacturer’s instructions from three biological replicates with pools of 7 mosquitoes each from the following groups: mosquitoes from Djougou phenotyped as resistant to pirimiphos-methyl (DP), alphacypermethrin (DA) or deltamethrin (DD); mosquitoes from Djougou unexposed to insecticides (DU); mosquitoes from Bassila phenotyped as resistant to pirimiphos-methyl (BP) or deltamethrin (BD); mosquitoes from Bassila unexposed to insecticides (BU) and mosquitoes from the susceptible Kisumu strain (KIS). The Agilent 4200 TapeStation was used to measure the RNA concentration and integrity. Ribosomal RNA was depleted using the Ribo-Zero™ Magnetic Core Kit and Ribo-Zero™ rRNA Removal kit (Illumina, USA). Library preparation was carried out using the ScriptSeq v2 RNA-Seq Library Preparation Kit (Epicenter, Illumina) according to the manufacturer’s instructions. Libraries were purified using Agencourt AMPure XP beads (Beckman Coulter, USA). The quantity and size distributions of the libraries were assessed using the Agilent DNA ScreenTape assay. Equimolar amounts of each library were pooled and sequenced (2 × 125 bp paired-end) on an Illumina HiSeq 2500 sequencer, using v2 chemistry. Sequencing was performed at the Biotechnology Core Facility at CDC, Atlanta, USA.

### RNA-Seq data quality control and mapping

Quality control was performed on the raw demultiplexed paired end sequencing reads obtained from the sequencing center using FastqC [54]. Reads from lane 1 and lane 2 were concatenated and trimmed to remove polyG tails, polyX at the 3′ ends, bases that did not meet the minimum quality score of 20 and paired reads where one or both were shorter than 50 bp using Fastp [55]. The trimmed reads (R1/R2) were aligned to the *An. gambiae PEST* reference genome (GenBank assembly identifier = GCA_000005575.2), directly downloaded from Vectorbase (release48) using ‘subjunc’, which is part of the subread aligner package, version 2.0.1 [56] with default settings. The alignments were filtered to remove low quality mapping reads (< 10) and sorted using SAMtools (v.1.10) [57].

FeatureCounts from the subread package version 2.0.1 [56, 58] was used to count tags that overlapped the coding sequence (CDS) features by at least 1 bp in the sense orientation of the gene set AgambiaePest (structural annotation version = AgamP4.13). Tag counts represent the number of sequence reads that originated from a particular gene. The higher the number of counts, the more reads associated with that gene, and the assumption that there is a higher level of expression of that gene in the sample. To identify main sources of variability in the dataset, Principal Component Analysis (PCA) was performed and visualized using ‘ggplot2’ in R.

Differential gene expression analysis was performed using the package EdgeR version 4.1.1 [59]. Thus, to remove the effect of noise and genes with very low expression, only genes where at least a tag count of 50 or more was obtained across all libraries were considered. CalcNormFactors, a function of the edgeR package that uses the TMM (trimmed mean M-values) method, was used to normalize the number of tags between samples, by finding a set of scaling factors for the library sizes that minimize log-fold changes between samples for most genes. DEGs between the different comparisons were selected after multiple testing using the decideTests function, of the limma package [60]. An absolute fold-change > 2 and FDR (false discovery Rate) ≤ 0.01 were used as statistical cutoffs to tag a gene as a DEG.

Comparisons were made between i) resistant field mosquitoes and laboratory susceptible mosquitoes (R-S): (DA vs KIS; DD vs KIS; DP vs KIS; BD vs KIS; BP vs KIS); ii) unexposed field mosquitoes and laboratory susceptible mosquitoes (C-S): (DU vs KIS; BU vs KIS) and iii) resistant field mosquitoes and unexposed field mosquitoes (R-C): (DA vs DU; DD vs DU; DP vs DU; BD vs BU; BP vs BU). R-S and C-S comparisons were made following the assumptions that constitutive resistance genes would be differentially expressed between both bioassay survivors and the unexposed mosquitoes when compared to the susceptible strain. The R-C comparison was made to detect gene expression that may be induced due to insecticide exposure. In addition, insecticide-specific comparisons were made (DD vs BD and DP vs BP) to detect DEGs associated with the same insecticide across the different mosquito populations.

### Detection of non-synonymous target site mutations

Target site mutations were detected from the variant calling analysis. Sorted bam files of the three replicates for each sample (DA, BD, DD, BP, DP, KIS) were used. Read coverage was counted at each genomic position to provide genotype likelihoods using the ‘mpileup’ methods of BCFtools [57, 61]⁠. Single nucleotide variants (SNVs) were then detected using bcftool ‘call’ and the resulting vcf file was filtered with vcfutils.pl [57, 61]⁠.

For each population, the frequencies of known point mutations in the *Ace-1* gene (G280S) and in the *kdr* gene (A1125V, A1746S, A1934V, D466H, E1597G, F1920S, I1527T, I1868T, I1940T, K1603T, L995F, L995S, M490I, N1575Y, P1874L, P1874S, T791M, V1254I, V1853I, V402L, and V1853I) were calculated.

### Gene ontology enrichment analysis

Gene ontology enrichment analysis was used to classify the differentially expressed genes based on specific biological functions using Goatools [62]⁠. This approach allows enrichment analysis to indicate which molecular functions, biological processes or cellular components were overrepresented (enriched) in a DEG list compared to an annotated list of the whole genome of *An. gambiae* obtained from blast2go [63] (Additional file 15)⁠. The GO term enrichment analysis of up- and downregulated genes was carried out using the Database for Annotation, Visualization, and Integrated Discovery (DAVID) [64, 65]. The *P*-values used to assess significantly enriched GO terms associated with the DEG list were calculated based on Fisher’s exact test and corrected by the Benjamini-Hochberg multiple testing correction method. Thus, an FDR adjusted P-value < 0.05 was used to report significantly enriched GO terms from the list of DEGs (Additional file 12). Visualization of the enriched GO terms was performed using the package ‘ggplot2’ following the protocol of Bonnot and others [66]⁠.

### RNAseq data validation using quantitative PCR

To validate the RNA-seq data, a set of six genes (SG7, CYP9K1, CYP6P3, COEJHE5E, RPS7, Actinc5) was used. RNA from three replicates of samples resistant to alphacypermethrin, deltamethrin or pirimiphos-methyl from Djougou (same batch of mosquitoes used for RNA sequencing) was used to synthesize cDNA using the HighCapacity cDNA reverse transcription kit (Applied Biosystems) with oligo-dT20 (NEB), according to the manufacturer’s instructions. The primers used are listed in Additional file 16. Standard curves of Ct values for each gene were generated using a serial dilution of cDNA, allowing assessment of PCR efficiency. qPCR amplification was carried out on an Agilent Technologies Stratagene Mx3005P using PowerUp SYBR Green Master Mix (Applied Biosystems). cDNA from each sample was used as a template in a three-step program: 50 °C for 2 minutes denaturation at 95 °C for 10 minutes, followed by 40 cycles of 15 seconds at 95 °C, 1 minute at 60 °C and a final step of 15 seconds at 95 °C, 1 minute at 60 °C, and 15 seconds at 95 °C. The relative expression level and fold change (FC) of each target gene from resistant field samples relative to the susceptible lab strain were calculated using the 2 − ΔΔCT method [67] incorporated in Python script (https://github.com/dany-gaga/from_Ct_to_logFoldChange). Housekeeping genes encoding ribosomal protein S17 (RPS17; AGAP010592) and Actin5C (AGAP000651) were used for normalization. A Pearson correlation coefficient was computed to assess the statistical difference between log2Foldchange obtained from resistant against susceptible strain comparisons for both RNAseq and qPCR results. The coefficient was computed and plotted using the ‘ggscatter’ function from the ‘ggpubr’ package in R.

### Supplementary Information

**Supplementary Material 1.**

**Supplementary Material 2.**

**Supplementary Material 3.**

**Supplementary Material 4.**

**Supplementary Material 5.**

**Supplementary Material 6.**

**Supplementary Material 7.**

**Supplementary Material 8.**

**Supplementary Material 9.**

**Supplementary Material 10.**

**Supplementary Material 11.**

**Supplementary Material 12.**

**Supplementary Material 13.**

**Supplementary Material 14.**

**Supplementary Material 15.**

**Supplementary Material 16.**

## Data Availability

The dataset supporting the conclusions of this article is available at Sequence Read Archive (SRA) under the accession number PRJNA982704. Project link: https://www.ncbi.nlm.nih.gov/bioproject/PRJNA982704

## Abbreviations

IR: Insecticide resistance
ITNs: Insecticide-treated nets
IRS: Indoor residual spraying
DGE: Differential gene expression
FC: Fold-change
PCA: Principal component analysis
CPs: Cuticular proteins
CYPs: Cytochrome P450 genes
GSTs: Glutathione-S-transferases
COE: Carboxylesterases
SNPs: Single nucleotide polymorphisms
GOE: Gene ontology
BP: Biological process
MF: Molecular function
CC: Cellular component
NMCP: National Malaria Control Program
LLINs: Long-lasting insecticidal nets
SGPs: Salivary gland proteins
RPS17: Ribosomal protein S1
CDS: Coding sequence
GO: Gene ontology
DAVID: Database for Annotation, Visualization, and Integrated Discovery
CTAB: Cetyl trimethyl ammonium bromide
