# Supplementary figures and images for: Transcriptomic analysis of Anopheles gambiae from Benin reveals overexpression of salivary and cuticular proteins associated with cross-resistance to pyrethroids and organophosphates
Source: BMC Genomics. 2024 Apr 6;25:348. doi: 10.1186/s12864-024-10261-x (PMC10998338; doi:10.1186/s12864-024-10261-x)

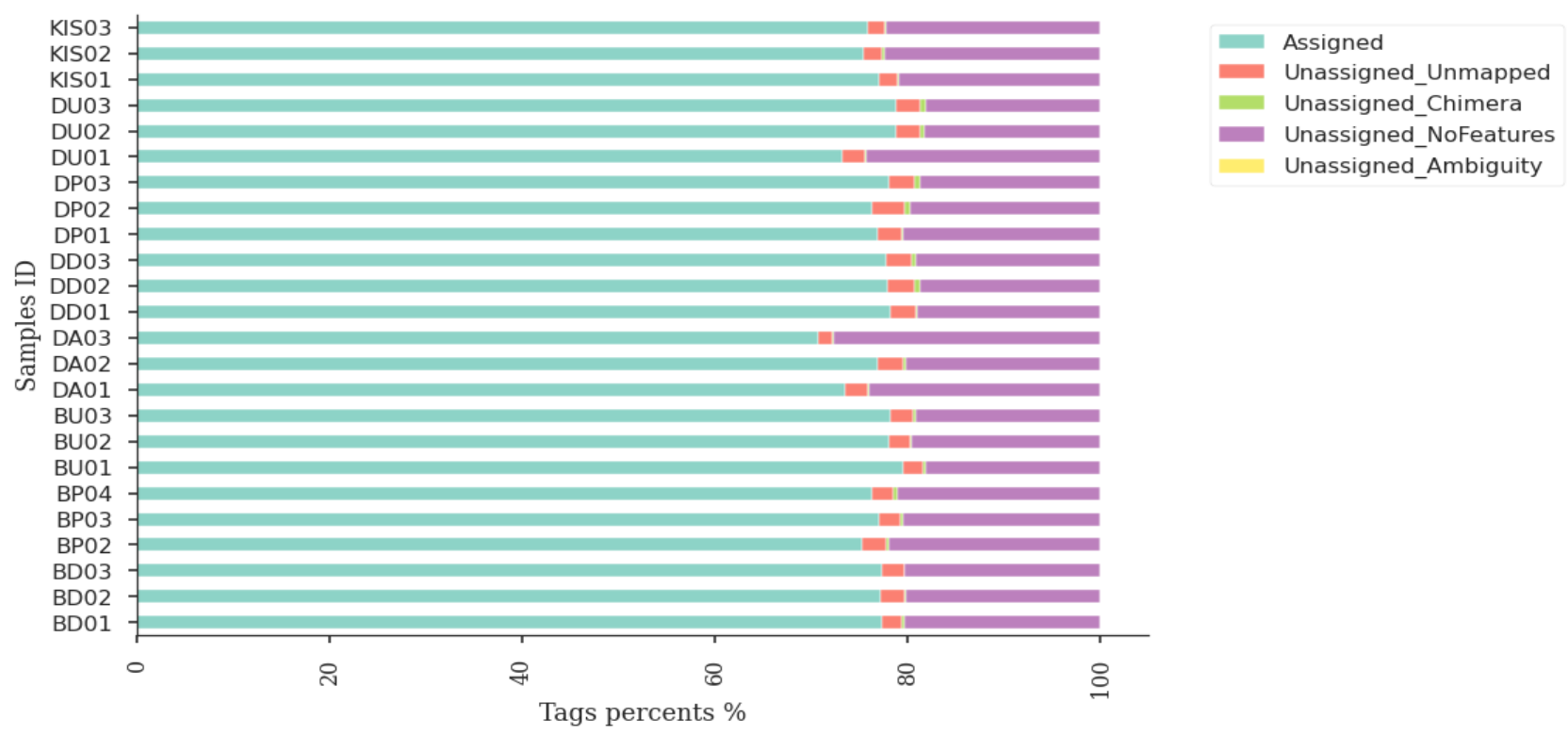

**Additional file 3:** Summary statistics of the read quantification

Supplement: Supplementary file 3 — Supplementary Material 3. [file 12864_2024_10261_MOESM3_ESM.pdf]
