# Supplementary material for: Transcriptomic analysis of Anopheles gambiae from Benin reveals overexpression of salivary and cuticular proteins associated with cross-resistance to pyrethroids and organophosphates
Source: BMC Genomics. 2024 Apr 6;25:348. doi: 10.1186/s12864-024-10261-x (PMC10998338; doi:10.1186/s12864-024-10261-x)
Supplement: Supplementary file 4 — Supplementary Material 4. [file 12864_2024_10261_MOESM4_ESM.pdf]

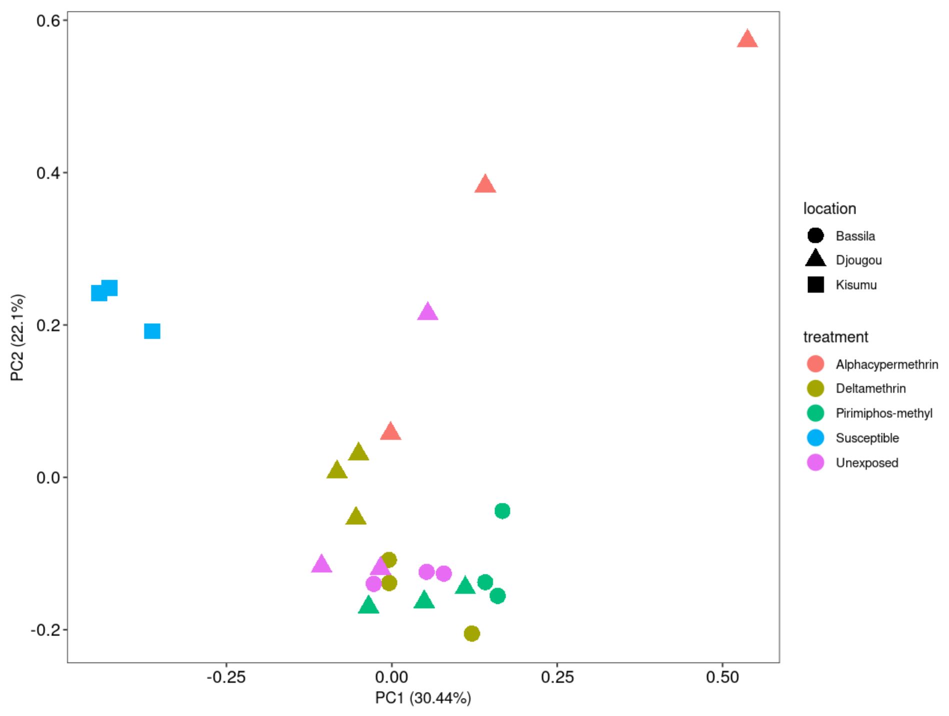

Additional file 4: PCA plot presenting an overview of the mosquitoes per location and treatment

The plot demonstrates a distinct separation between the susceptible strain Kisumu and the mosquitoes from Bassila and Djougou when exposed to the different insecticides alphacypermethrin, deltamethrin and pirimiphosmethyl.
