# Supplementary material for: Transcriptomic analysis of Anopheles gambiae from Benin reveals overexpression of salivary and cuticular proteins associated with cross-resistance to pyrethroids and organophosphates
Source: BMC Genomics. 2024 Apr 6;25:348. doi: 10.1186/s12864-024-10261-x (PMC10998338; doi:10.1186/s12864-024-10261-x)
Supplement: Supplementary file 6 — Supplementary Material 6. [file 12864_2024_10261_MOESM6_ESM.pdf]

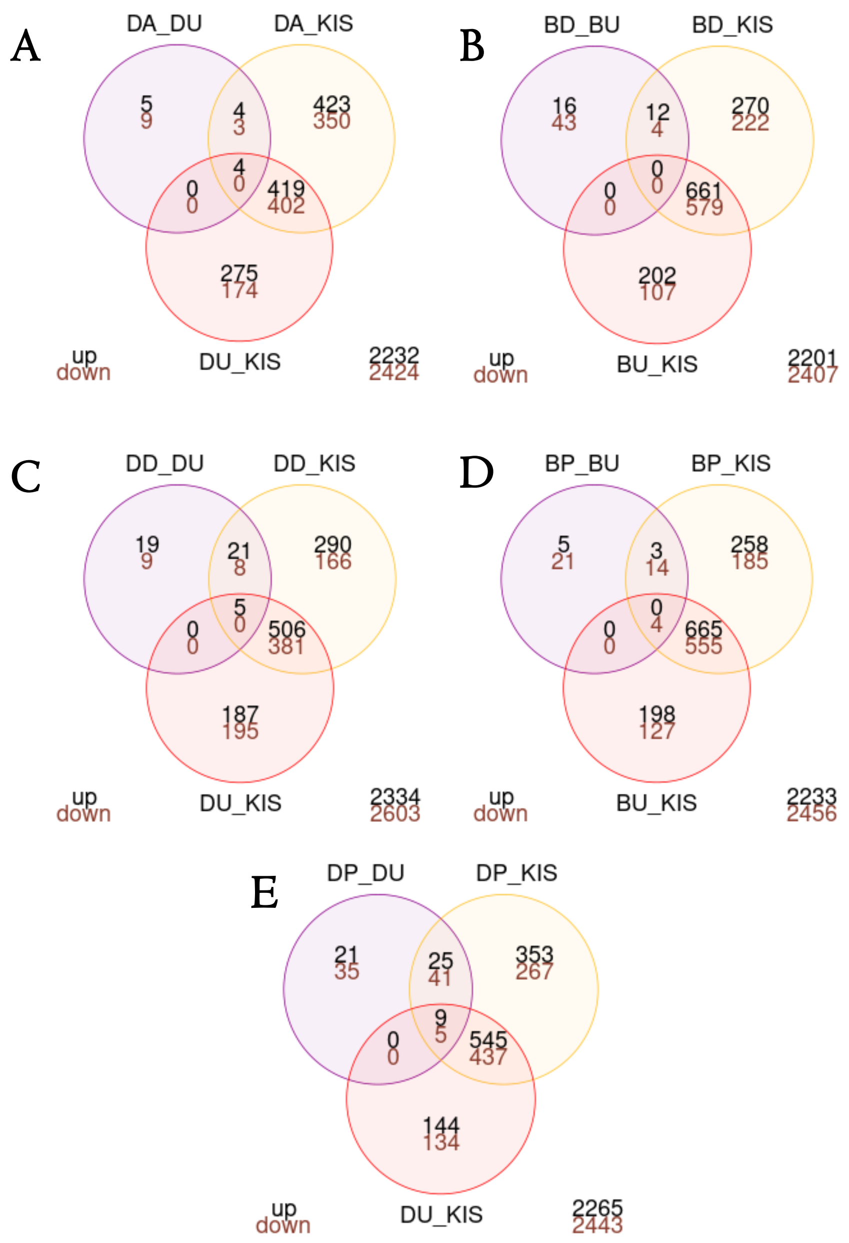

Additional file 6: Venn diagram showing differentially expressed genes among resistant, susceptible and unexposed mosquito populations. Panel A represents gene differentially expressed in Bassila when mosquitoes are resistant to deltamethrin; in B when they are exposed to pirimiphos-methyl; in C when mosquitoes from Djougou are exposed to deltamethrin; in D when exposed to pirimiphos-methyl and in E when exposed to alphacypermethrin. Each Venn diagram section shows the number of differentially expressed genes meeting each set of conditions and the p values were adjusted for multiple testing based on FDR 2.
