# Supplementary material for: Transcriptomic analysis of Anopheles gambiae from Benin reveals overexpression of salivary and cuticular proteins associated with cross-resistance to pyrethroids and organophosphates
Source: BMC Genomics. 2024 Apr 6;25:348. doi: 10.1186/s12864-024-10261-x (PMC10998338; doi:10.1186/s12864-024-10261-x)
Supplement: Supplementary file 13 — Supplementary Material 13. [file 12864_2024_10261_MOESM13_ESM.pdf]

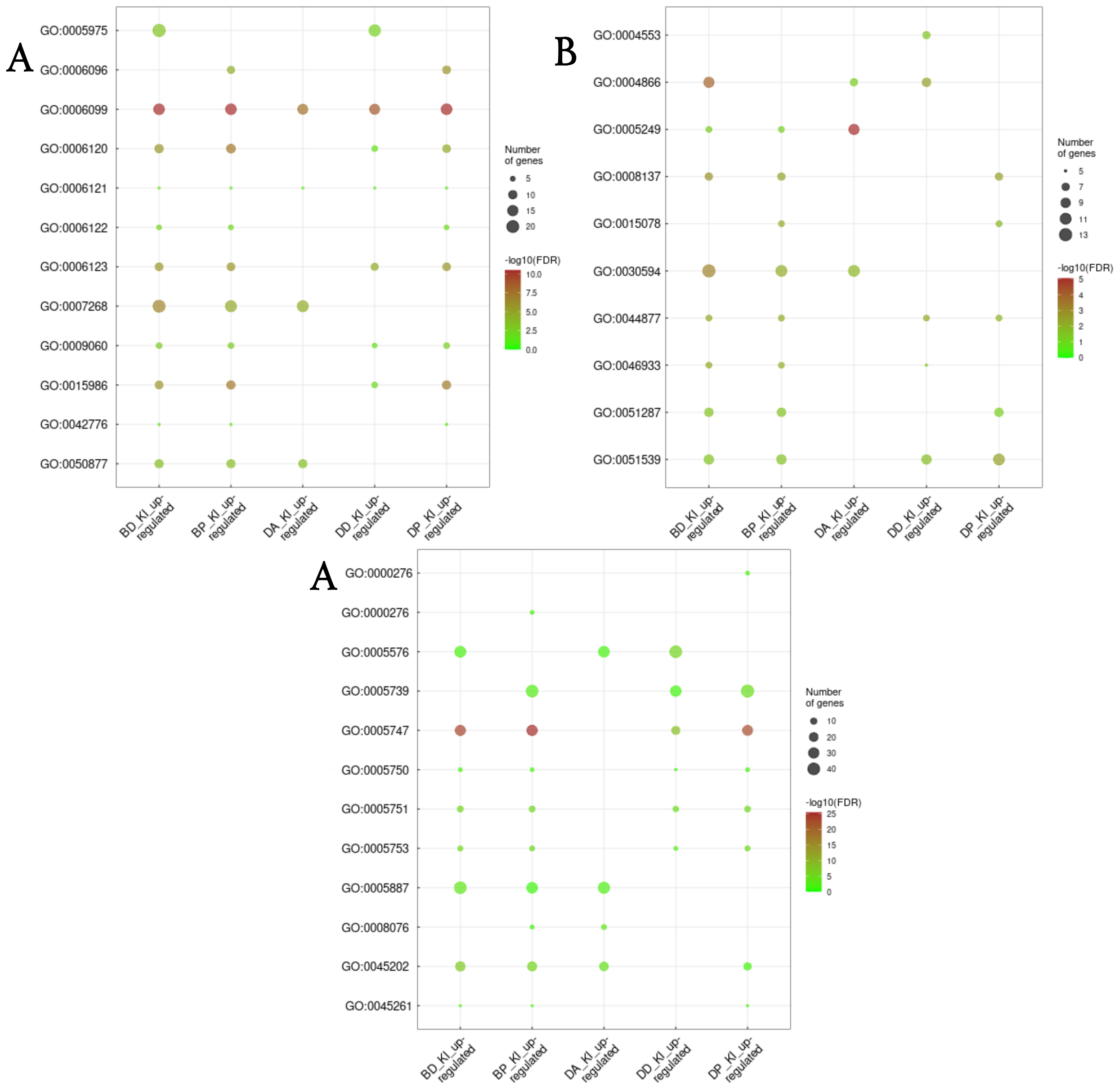

Additional file 13: Gene ontology enrichments Panel A shows enriched terms within biological process; B, the enriched terms in molecular functions and C, the cellular components.
