# Supplementary material for: Transcriptomic analysis of Anopheles gambiae from Benin reveals overexpression of salivary and cuticular proteins associated with cross-resistance to pyrethroids and organophosphates
Source: BMC Genomics. 2024 Apr 6;25:348. doi: 10.1186/s12864-024-10261-x (PMC10998338; doi:10.1186/s12864-024-10261-x)
Supplement: Supplementary file 14 — Supplementary Material 14. [file 12864_2024_10261_MOESM14_ESM.pdf]

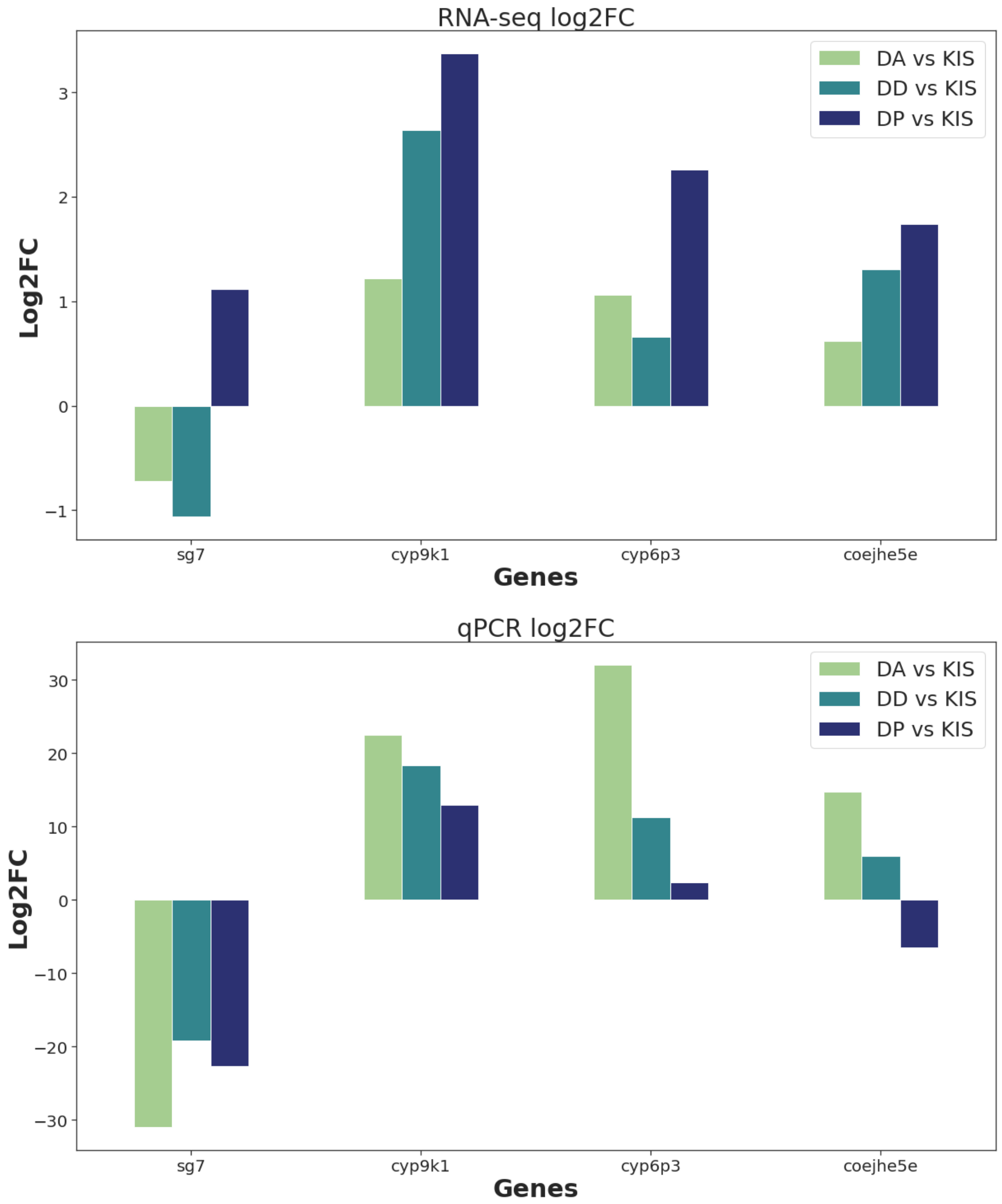

Additional file 14: Comparison of expression levels of DEGs measured by qRT-PCR and RNA-Seq Comparisons between mosquitoes exposed to alphacypermethrin (DA), deltamethrin(DD) and pirimiphos-methyl(DP) against Kisumu (KIS
